# Editorial: Role of NOD-Like Receptors in Infectious and Immunological Diseases

**DOI:** 10.3389/fimmu.2020.00923

**Published:** 2020-05-19

**Authors:** Christopher R. Lupfer, Paras K. Anand, Xiaopeng Qi, Hasan Zaki

**Affiliations:** ^1^Department of Biology, Missouri State University, Springfield, MA, United States; ^2^Department of Infectious Disease, Imperial College London, London, United Kingdom; ^3^Kunming Institute of Zoology, Chinese Academy of Sciences, Kunming, China; ^4^Department of Pathology, University of Texas Southwestern Medical Center, Dallas, TX, United States

**Keywords:** NLRP3, inflammasome, inflammation, NOD-like receptor, infection, immunological diseases

NOD-like Receptors (NLRs) are a large family of 22 intracellular proteins in humans. Although NLRs are structurally homologous to each other, they perform a diverse array of cellular functions and play key roles in the regulation of innate immune responses. In this Research Topic, our understanding of NLRP3 has received particular emphasis. This most studied NLR family member has defined roles in infectious, autoimmune and autoinflammatory diseases signifying a paramount need to understand regulatory mechanisms that modulate NLRP3 activity. An et al. reviewed the known roles for NLRP3 in a suite of cardiovascular diseases (CVDs) where therapeutic interventions targeting NLRP3 might be successful in the foreseeable future. A review of clinical trials included by An et al. highlights some promising new candidates for CVD such as canakinumab, and some old drugs (like statins and colchicine) being used to treat CVD whose mechanisms of action may include inflammasome inhibitory functions. Zahid et al. reviewed current drugs targeting NLRP3 as well as future avenues for therapeutic development. These include drugs that directly target NLRP3 to prevent oligomerization or interaction with ASC, targeting transcriptional regulation of NLRP3 inflammasome constituents, inhibition of gasdermin D-mediated pyroptosis, or blockade of inflammasome cytokines IL-1β or IL-18. As Zahid et al. pointed out, development of drugs that target only the undesired aspects of inflammasome activation, while leaving others intact, may allow for greater therapeutic benefits. Finally, Zhao and Zhao evaluated the vast literature describing the roles of NLRP3 during viral infection. Although much evidence supports the role for ion flux in NLRP3 inflammasome activation, Zhao and Zhao discuss the more recent findings that describe NLRP3 interaction with cytoplasmic RNA sensors, such as RNAse L and DHX33, to detect viral PAMPs and not just cellular DAMPs. Other groups have shown that NLRP9 similarly detects PAMPs through interactions with cytoplasmic RNA sensors (1). NLRC4 also detects PAMPs through interactions with NAIPs (2, 3). Taken together, these findings suggest that some NLRs may function as points of convergent signaling while other receptors display ligand specificity. Zhao and Zhao further recapped how viruses have evolved to inhibit the NLRP3 pathway. Viruses have been in the business of subverting immune signaling much longer than humans, perhaps this knowledge can help guide future development of therapeutics as discussed by An et al. and Zahid et al..

Three primary research articles in this Research Topic presented evidence for the role of NLRP3 in infectious disease. One novel study demonstrated that NLRP3 is a key player in the development of inflammation, but that inflammation is a double-edged sword. Liu et al. observed that Zika virus replicates in the kidney and induces the activation of the NLRP3 inflammasome. However, NLRP3-mediated signaling triggered unwanted overt inflammation resulting in kidney damage during Zika virus infection, which was associated with altered aquaporin expression in the kidney tissue. The other two primary research articles showed that infections caused by different bacterial serotypes can activate different or additional inflammasome pathways than those initially reported. *E. coli* and *V. cholerae* can activate the non-canonical NLRP3 inflammasome (4). However, Verma et al. observed that uropathogenic *E. coli* infections in human patients induce the expression of NLRP3 and NLRC4 inflammasome components as revealed by the presence of active caspase-1 in patient samples. Furthermore, the CFT073 strain of *E. coli* could induce formation of the NLRC4 inflammasome in addition to the NLRP3 inflammasome. In a different model, Mamantopoulos et al. show that El Tor biotype *V. cholerae* activates the canonical, caspase-11 independent, NLRP3 inflammasome through its hemolysin (hlyA) toxin. They further demonstrated that El Tor biotype *V. cholerae* can activate the Pyrin inflammasome, though to a lesser degree. Overall, these primary research reports call into question the function of some forms of inflammation in fighting infectious disease when it often results in excessive tissue damage. They also point to the necessity for more research into the scope of pathogen recognition for specific NLR pathways. Different bacterial isolates or serotypes should be examined for differences in NLR inflammasome specificity.

Although NLRP3 plays critical roles in the pathogenesis of many diseases and is a highlight of this Research Topic, two articles summarized the role of other NLRs in disease. Martínez-Torres and Chamaillard eloquently discussed the importance of post translation modifications, specifically ubiquitination, in the NOD1 and NOD2 signaling pathways and how these modifications modulate inflammatory responses and disease pathogenesis. A second review by Nagai-Singer et al. focused on NLRX1 and provided a significant overview of its unresolved functions. It has been shown by different groups that NLRX1 has diverse functions including either induction or suppression of inflammatory responses, cell death regulation, autophagy and more. This review attempted to unify those findings and highlighted the fact that NLRX1 exerts different physiological functions depending on cell types, disease models, and methodologies (RNAi vs. genetic ablation). Clearly, more research is needed to clarify the functions of NLRX1 and specifically address the possibility that it may have different or complementary functions under different conditions.

Overall, the articles in this research collection contributed to a broad review of our current knowledge in the field of NLR biology related to infectious and inflammatory diseases. Others have specifically expanded our understanding of NLR signaling pathways with their functions during different infectious and inflammatory diseases. Moreover, the results, hypotheses, and postulations made by the authors of the articles in this collection provide exciting avenues of future research, namely, the development of viable therapeutics to target NLRs and their downstream signaling pathways, the need to more carefully examine NLR receptor specificity for specific pathogens, and the need for more detailed characterization of the molecular mechanisms involved in NLR signaling and activation. We hope this collection is both informative in its content and evocative in fostering future research in the area of NLR biology.

## Author Contributions

CL wrote the manuscript and PA, HZ, and XQ provided conceptual insight and editing of the manuscript.

## Conflict of Interest

The authors declare that the research was conducted in the absence of any commercial or financial relationships that could be construed as a potential conflict of interest.
